# An approach for improving the quality of country-level TB modelling

**DOI:** 10.5588/ijtld.21.0127

**Published:** 2021-08-01

**Authors:** C. F. McQuaid, M. C. Clarkson, M. Bellerose, K. Floyd, R. G. White, N. A. Menzies

**Affiliations:** 1 TB Modelling Group, TB Centre and Centre for Mathematical Modelling of Infectious Diseases, Department of Infectious Disease Epidemiology, London School of Hygiene & Tropical Medicine, London, UK; 2 Department of Global Health and Population, Harvard TH Chan School of Public Health, Boston, MA, USA; 3 Global TB Programme, World Health Organization, Geneva, Switzerland; 4 Center for Health Decision Science, Harvard TH Chan School of Public Health, Boston, MA, USA

**Keywords:** benchmark, reporting, review, tuberculosis, policy, strategic planning

## Abstract

Mathematical modelling is increasingly used to inform budgeting and strategic decision-making by national TB programmes. Despite the importance of these decisions, there is currently no mechanism to review and confirm the appropriateness of modelling analyses. We have developed a benchmarking, reporting, and review (BRR) approach and accompanying tools to allow constructive review of country-level TB modelling applications. This approach has been piloted in five modelling applications and the results of this study have been used to revise and finalise the approach. The BRR approach consists of 1) quantitative benchmarks against which model assumptions and results can be compared, 2) standardised reporting templates and review criteria, and 3) a multi-stage review process providing feedback to modellers during the application, as well as a summary evaluation after completion. During the pilot, use of the tools prompted important changes in the approaches taken to modelling. The pilot also identified issues beyond the scope of a review mechanism, such as a lack of empirical evidence and capacity constraints. This approach provides independent evaluation of the appropriateness of modelling decisions during the course of an application, allowing meaningful changes to be made before results are used to inform decision-making. The use of these tools can improve the quality and transparency of country-level TB modelling applications.

## Abstract (FR)

Les programmes nationaux de lutte contre la TB ont de plus en plus recours aux modélisations mathématiques pour éclairer l’établissement du budget et la prise de décision stratégique. Malgré l’importance de ces décisions, il n’existe actuellement aucun mécanisme permettant de vérifier et confirmer la pertinence des analyses de modélisation. Nous avons développé une méthode de référenciation, rapportage et examen (de l’anglais, *benchmarking, reporting and review* [BRR]), avec outils connexes, pour une analyse constructive des modélisations liées à la TB à l’échelle d’un pays. Cette approche a été testée avec cinq applications de modélisation et les résultats de cette étude ont été utilisés afin de corriger et de finaliser l’approche. L’approche BRR comprend : 1) des points de référence quantitatifs avec lesquels les hypothèses et résultats des modélisations peuvent être comparés, 2) des modèles de rapports standardisés et des critères d’examen, et 3) un processus d’examen en plusieurs étapes faisant remonter les informations aux modélisateurs pendant l’application du modèle, ainsi qu’une évaluation récapitulative une fois la modélisation terminée. Pendant l’essai pilote, l’utilisation d’outils a donné lieu à d’importantes modifications des approches de modélisation. L’essai pilote a également permis d’identifier des problèmes qui sortent du champ d’application d’un mécanisme d’évaluation, tels qu’un manque de données probantes empiriques et des contraintes de capacité. Cette approche fournit une évaluation indépendante de la pertinence des décisions de modélisation pendant l’application du modèle, permettant d’apporter des modifications significatives avant d’utiliser les résultats pour éclairer la prise de décisions. L’utilisation de ces outils peut améliorer la qualité et la transparence des modélisations liées à la TB à l’échelle d’un pays.

The use of mathematical modelling to support TB policy-making has been encouraged by major funding agencies and adopted by many high-burden countries. These quantitative planning exercises are undertaken to prioritise proposed interventions, estimate resource needs and support funding applications. Over the past 2 years alone, the number of countries using TB modelling to develop national strategic plans or inform funding applications has doubled from around 20 to 40.^1^ The increased use of modelling has also highlighted differences in the findings of individual modelling studies. Model comparison exercises for TB^2^–^4^ and other diseases^5^–^8^ have revealed that differences in results occur even when models evaluate the same policy alternatives in the same setting. In some cases, these differences are of sufficient magnitude to influence the policy conclusions drawn. This variability threatens to undermine confidence in modelling for decision-making. There is also concern that modelling for new policy options may be overly optimistic if it does not anticipate the implementation challenges encountered during roll-out.^9^ Major uncertainties in the empirical evidence base^10^ mean that different models should not always be expected to align, but there is currently no mechanism to ensure the quality of modelling applications, and it is possible that choices about model structure and inputs could be improved through independent review.

Academic journals have a long tradition of independent peer-review to assess the quality of published work. However, these peer review mechanisms are not designed to test the modelling for country-level decision-making. Instead, modelling is typically subjected to peer-review after country-level decision-making is complete, if at all. Given the iterative nature and hard deadlines of country planning processes, traditional journal review is therefore unlikely to provide appropriate and timely input, as decisions will already have been made by the time feedback has been received. In addition, such review tends to focus on the technical quality of the work and how it is communicated as an academic manuscript,^11^ and less attention is given to the process of stakeholder engagement and how modelling results and limitations are communicated to this key group.

In 2018, the WHO and the TB Modelling and Analysis Consortium (TB MAC) published guidance for mathematical modelling used to support country-level TB decision-making, consolidating inputs from a wide range of stakeholders.^12^,^13^ The guidance describes principles and good practices for country-level modelling, but does not describe how these principles should be operationalized. This encouraged individual modelling groups to ensure that good practices were followed, but without a clear mechanism to confirm that this had occurred. To respond to this need, we have developed an approach designed to evaluate the quality of individual modelling applications and strengthen the incentives for quality and transparency of these studies. Here we describe the development and piloting process, and the finalised approach.

## DESIGN CONSIDERATIONS

We developed the benchmarking, reporting and review (BRR) approach to reveal where a given modelling application is inconsistent with existing evidence or modelling best-practices,^13^ and provide feedback to modellers that can be used to revise the analysis. Supplementary Table S1 describes proximal, intermediate and final objectives of the approach. Several considerations for the design of the approach were highlighted during conceptualisation. First, due to the importance of contextual factors in determining the appropriateness of modelling methods, the approach should evaluate the adequacy of modelling as used in a specific application, rather than attempting a global assessment of model validity. Second, the review should consider not only the technical quality of modelling, but also the level of engagement with stakeholders within the modelling application, responsiveness to country needs and clarity of dissemination, as these are equally important in determining the value of modelling for decision-making. Third, the review should provide feedback to modellers during the course of the application, which can be incorporated before analyses are finalised. Fourth, given the range of reasonable modelling approaches that could be adopted, the results of the review should not be prescriptive, and modellers should be able to provide a response justifying their chosen approach. Fifth, modelling applications require a range of expertise, including TB programme strategy, epidemiological modelling and economics, and the reviewer(s) should be able to cover these domains.

## DEVELOPMENT PROCESS

An initial approach was prepared by a small writing committee, and further developed by a working group of modellers, economists, TB programme experts and donor representatives. This approach was presented at the 2018 WHO/TB MAC annual meeting in Washington DC, USA,^14^ where input was invited from a wider stakeholder group that included modelling groups, economists, country stakeholders, funders, advocates and other technical experts. The approach was piloted in a sample of five country-level modelling applications, and additional input was obtained at the 2019 WHO/TB MAC annual meeting in Istanbul, Turkey.^15^

## PILOT STUDY

We piloted the BRR approach in five country-level modelling applications conducted between 2018 and 2020 for high TB burden countries using modelling technical assistance to develop funding requests for one of several funding agencies. These included a variety of settings (Kenya, Bhutan, Indonesia, Mongolia, Myanmar); several funding agencies (The Bill and Melinda Gates Foundation, the Global Fund, the United States Agency for International Development); and different modelling groups (Imperial College London, the Australian Tuberculosis Modelling Network and the London School of Hygiene & Tropical Medicine). Policy questions addressed included the prioritisation and cost-effectiveness of interventions for strategic planning, resource allocation, the development of targets and programmatic strategy required to reach targets. For each review, we engaged three independent reviewers, with expertise in epidemiological modelling, economics and TB programme strategy, respectively. After each completed review, we conducted interviews with modellers and reviewers to assess their experience with the review process (see Supplementary Data). This feedback was used to revise and finalise the approach.

Ethical approval was not required for the study as it did not involve human participants.

## PILOT STUDY EVALUATION

Modellers participating in the pilot study reported that the BRR approach provided a structure that added clarity and standardisation to the review, and that the review feedback led modellers to reconsider modelling approaches and how these were communicated. Examples of these changes included removing intervention scenarios for which empirical evidence was weak, and more explicit description of uncertainties and costs. Participants considered the BRR to have improved the transparency and quality of modelling applications, and also suggested modified modelling approaches they would apply to future applications. Participants reported the templates and benchmarks were detailed and useful, and the process provided sufficient flexibility and opportunity to raise disagreements. However, several participants reported that the time required to participate in the review could conflict with the timing of the modelling application. Participants noted that competing priorities and tight deadlines of the country planning process meant that reviews frequently needed to be completed urgently, and modellers had limited time to incorporate feedback. In one application, stakeholders required the modelling to be finalised before the final stages of review could be completed. Detailed feedback is included in Supplementary Data. The approach was revised based on the results of this pilot, including revisions to the text of individual forms and streamlining of the review process to better accommodate tight deadlines. The final approach is described below.

## THE BENCHMARKING, REPORTING, AND REVIEW APPROACH

The BRR approach comprises 1) quantitative benchmarks describing features of TB natural history, epidemiology, health services, and costs; 2) a standard format for reporting modelling methods for review, and additional templates to standardise the review process; and 3) a standard process for external review. Materials for implementing this approach are provided in Supplementary Data with an overview of the approach shown in the Figure.

**Figure:**
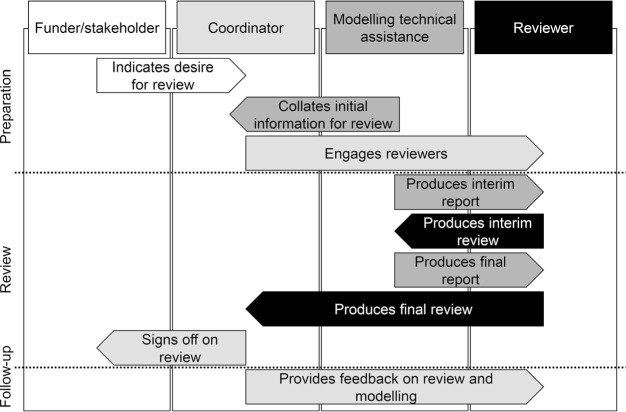
Flowchart of a typical review process, identifying actors and contact points in the review process. Different shading and columns indicate different actors in the review process. Arrows indicate the actor responsible for a particular step (arrow origin column) and the recipient (arrow destination column).

### Benchmarks

The benchmarks describe features of TB epidemiology and economics about which assumptions need to be made as part of the modelling application, and where independent evidence is available to judge the appropriateness of assumptions. Table 1 lists the major domains covered by these benchmarks. The benchmarks are divided into epidemiological and economic benchmarks, including general benchmarks, assumed to apply to all settings (for example, assumptions around TB natural history in immunocompetent adults); and country-specific benchmarks, where the benchmark value will vary between settings and over time (for example, modelled TB burden estimates could be compared to published values for the same country and year). Modelling assumptions can be compared to these benchmarks to assess their appropriateness for a given policy question and country context. The benchmarks also include a standard set of modelling results and projections for which no formal comparison value may exist (such as future epidemiological trends in the base-case modelling scenario), but which aid interpretation of the model results. The full set of benchmarks is provided in the Supplementary Data.

**Table 1:** Domains covered by modelling benchmarks ^*^

Benchmark area	Description
General epidemiological benchmarks	Describe general features of TB epidemiology, and are assumed to apply to most settings in which TB is being modelled to evaluate policy/intervention options. Unless otherwise stated, benchmarks apply to the HIV-negative general population. Disease definitions (active TB, latent TB) follow standard definitions described by the WHO, as operationalised in the model. For models that provide a range of results (stochastic models or probabilistic analyses) benchmarks should be compared to the point estimate (mean, median) reported from the model
Country-specific epidemiological benchmarks	Describe country-specific features of TB epidemiology. Modellers can make comparisons with the series of estimates most appropriate to their estimation task; possible options are shown below the table. Comparison values may be subject to estimation error, and an exact match is not required
Country-specific economic benchmarks	Describe features of TB programme resource utilisation that are assumed to be country-specific. Modellers can make comparisons with the data/estimates most appropriate to their estimation task
Additional standard outputs	Describe features of TB epidemiology and programme performance for which no benchmark is provided, but which are useful for interpreting model assumptions and results
Policy projections	Describe modelled epidemiological outcomes

* Full tables of benchmarks are provided in the reporting templates.

Although the benchmarks are intended to apply to most modelling scenarios, it is possible that specific modelling assumptions or results may deviate from these benchmarks due to unique features of the setting or population of interest. In this situation, modellers are not expected to modify their model to meet the benchmark, but instead to note the discrepancy and provide a satisfactory justification to explain the difference.

### Reporting format

Standard reporting templates are used to summarise key features of the modelling application for the purpose of review. Table 2 lists major domains covered by these templates, which include quantitative outcomes (including the benchmarks described above), a description of the modelling context and evaluation questions addressed, and key features of the modelling process. Modellers can also provide additional documentation (such as published papers or technical reports). Standardised templates are also provided for reviewers to record their review report. The templates are designed to be sufficiently general in nature to apply to all modelling applications, with clear instructions for what to do for optional sections. These templates are provided in the Supplementary Data.

**Table 2:** Summary of the final reporting domains *

Reporting domain	Reporting areas
Evaluation question	What is the primary research question for modelling? What is the primary audience for modelling results? What is the population being modelled, and are there sub-populations of particular interest? What policy alternatives are compared, and how were these identified? What outcomes are used to summarise health or epidemiological effects of policy alternatives? What type of economic analysis is being conducted, and what are the primary metrics used to report economic results? How are optimal policies chosen?
Process	Which stakeholders (local partners, funders, technical agencies or others) are participating in the modelling application? What activities are being undertaken to support local capacity building or institutionalisation? Are there any conflicts of interest (including the review process, if relevant)?
Results	What are the main findings and policy recommendations of the modelling? What are the major uncertainties or untested assumptions of this modelling? How were these limitations presented to decision-makers? What are the major threats to success of the novel policies examined? What is the most urgent or important research needed to confirm these findings? How will these modelling results be used in the policy process?
Benchmarks	Are results consistent with modelling benchmarks and other relevant comparison data? If there are deviations, how should these be interpreted? Are other steps being taken to validate the model? What uncertainty and sensitivity analyses are conducted, and what conclusions are drawn from these for policy recommendations? Describe the technical specifications of the economic analysis. Is empirical evidence available to support assumptions around the magnitude of changes in intervention coverage, quality or effectiveness, by intervention? Is there a more detailed model report that provides technical details of the model approach, including model structure, parameterisation, cost estimates or functions, application setting and results?

* Reporting and review templates for each stage of review are provided in the Supplementary Data.

### External review

The Figure provides a flow diagram of a typical review process, including the actors involved and steps taken to complete the review. The review itself is divided into two stages, and it is important to develop a shared timeline for the process as soon as possible to ensure that the review fits the requirements of the modelling application. The first-stage review evaluates initial results and is timed so that feedback can be used to adjust modelling decisions, if deemed appropriate by the modellers. The second-stage review considers finalised modelling results and provides a summary appraisal of the strengths and weakness of the modelling application. Supplementary Table S2 lists the high-level criteria against which independent external reviewers (not affiliated with the modelling team, funder or stakeholders) evaluate the modelling application, based on principles for good modelling practice described in the WHO Guidance for Country-Level TB Modelling.^13^ For each stage of review, the modelling team is expected to complete the reporting template, which is then sent to the reviewers to complete the review template and return the review. A discussion between the modelling team and reviewers may then be held to allow questions and clarifications. In the second-stage review, any unresolved areas of disagreement are noted and included in the final review report, including the recommended change and the response from the modelling team. The reviewers and the modelling group are asked to sign off on the completed final review and response, which is included in the final report. This report is shared with a list of recipients agreed at the start of the review. In situations where external review is not possible, modellers can undertake self-review using the BRR materials and compare modelling results to the benchmarks.

## DISCUSSION

The BRR approach represents a first attempt, outside of peer review of an academic article, to establish a routine peer-review process for mathematical modelling as part of country-level disease control strategy development. This approach establishes a platform for discussions between modellers and reviewers, enabling a constructive review of the modelling application. It can identify and resolve any areas where the application is inconsistent with existing evidence or best-practice modelling approaches, strengthening the evidence used to support decision-making. This approach does not attempt to draw broad conclusions about the validity of the models being used, but rather focuses on the appropriateness of modelling for a specific setting and policy question. For this reason, the approach is intended to complement periodic multi-model comparison studies^16^ (as have been conducted for TB,^2^–^4^ HIV,^5^,^8^ malaria,^7^ vaccine-preventable diseases,^6^,^17^ and COVID-19^18^), which allow direct comparisons between models but cannot address all the context-specific factors relevant to a given modelling application. While the approach is designed for routine use there may be some modelling applications where external review is not needed, such as small updates to earlier models. The decision to undertake external review will ultimately be made by the stakeholders in a given modelling application.

In a pilot study of the BRR approach conducted alongside five country-level modelling applications, participants reported that the reviewer feedback prompted improvements in the approaches taken to modelling by identifying intervention scenarios with insufficient empirical support. Modellers also reported that the process provided feedback to improve modelling methods and increased stakeholder confidence in the findings, without being overly prescriptive. However, issues with the coordination and timing of the review were reported. The stop-start nature of the country-level planning processes, combined with tight deadlines, meant that it was difficult at times to provide meaningful review in a way that could be incorporated into the modelling. Because of this experience, the approach was revised and streamlined. This pilot study provides initial evidence on how independent review can play a constructive role in country-level modelling applications, but additional steps to evaluate the impact of the BRR approach will be needed as it is used more routinely. It is expected that the BRR approach will require periodic revision as the role of modelling to support country strategic planning evolves over time. In particular, as national strategic plans are more directly linked to Global Fund funding requests, this will have a knock-on effect on the requirements of modelling, particularly around timing.

The development and piloting process revealed several challenges for country-level TB modelling beyond the scope of the BRR approach. First, a lack of high-quality empirical evidence was mentioned by a number of pilot study participants, limiting the ability of models to accurately represent complex scenarios or novel interventions. Second, as the approach only assesses a single modelling application at a time, it does not directly assess the variation in results possible with different models, yet this is still a concern in the field. Third, human capacity constraints and staff turnover, for both modelling groups and in-country stakeholders, can be a challenge to continuity and process improvement. These issues echo problems identified in the development of earlier guidance,^12^ and will likely require solutions beyond the BRR approach.

Increasing the alignment of modelling applications with standardised planning processes – most notably those following the WHO-endorsed People-Centred Framework for TB Programme Planning and Prioritization^19^ – will help ensure that modelling evidence is integrated into planning cycles, that routinely collected data can inform modelling applications and that models are better prepared to answer new policy questions. Institutionalisation of the review process within a funding body or major technical agency would be beneficial, providing a unit to identify modelling applications for review and coordinate these reviews. This institutionalisation would provide continuity for the review mechanism and better align the incentives modellers face. In parallel to these efforts to institutionalise external reviews for modelling applications, new research is needed to strengthen the empirical evidence base for modelling and investigate other sources of variation in modelling results.

## CONCLUSION

The BRR approach is designed to improve the quality and usefulness of TB modelling used for country-level decision-making. If routinely implemented, this approach will strengthen the incentives for high-quality modelling work, identify common areas where modelling methods or the empirical evidence base need to be strengthened, and stimulate the progressive improvement of TB modelling as a tool to inform country-level TB strategy.
